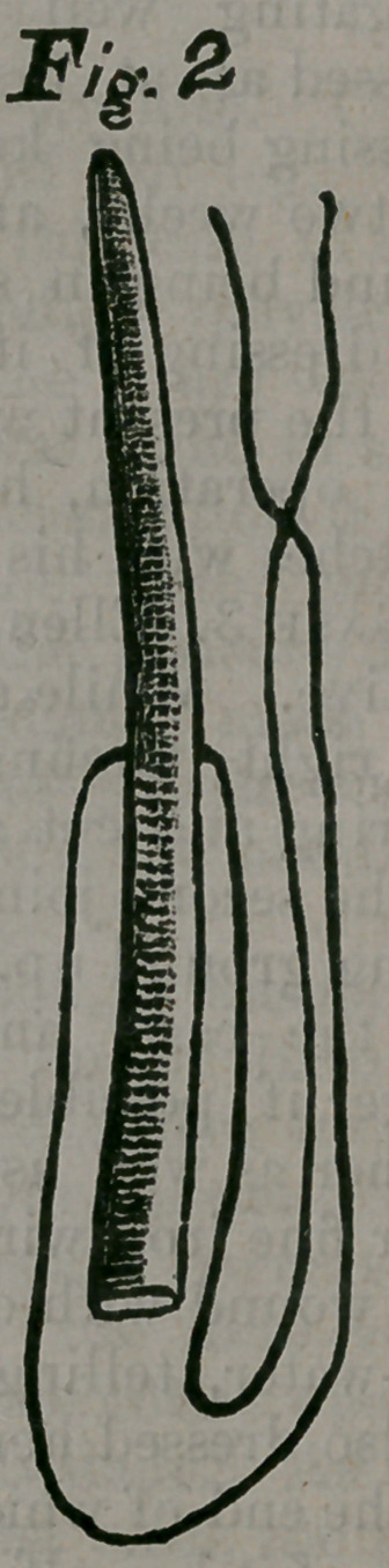# The Pith of Dried Cornstalk as a Uterine Tent

**Published:** 1878-09-20

**Authors:** W. T. Goldsmith

**Affiliations:** Atlanta, Ga.


### THE PITH OF DRIED CORN-STALK AS A UTERINE TENT.

By W. T. Goldsmith, M.D., Atlanta, Ga.

The author prepared an article, which was read before the Georgia Medical Association, upon the use of "The Pith of the Cornstalk as a Uterine Tent." By request of the managing editor, I offer a brief synopsis of the practical points of the paper. In the paper referred to, I say:

I have the pleasure of bringing before the profession a new candidate for their consideration as a uterine tent. It is the pith of the dried cornstalk. It may, or may not, have advantages over other materials manufactured into tents. I will permit the profession to determine this matter. To my mind, there are many points of superiority. These points will be developed as I proceed with the reading of this paper. I will, however, pause long enough to show the ease and rapidity with which they can be made. You take a joint of the dried stalk; strip it of its cuticle, and compress the pith, slowly and firmly, between the thumbs and index fingers. You see how compression, made in this way, diminishes its bulk. By continued pressure, you easily reduce it to four or five times less its original size. You can compress it to any intermediate size; or, it may be used without compression to carry medicaments to the interior of the uterus. Because of its ready compression to any desired bulk, you have the tent entirely under your control. Slight compression will give you moderate dilating power. Compress it as much as possible, and you can get a dilating power equal to the sea-tangle or sponge. You may compress the pith first, and afterwards, with a sharp knife, trim to the desired length and size; or, you may first cut the pith to the size you wish it to dilate, and then compress it for easy introduction. Any curve may be given the tent by selecting a piece of pith that has been curved in its growth. The pith absorbs fluids readily, and, when compressed, expands by such absorption to its original size.

The wood-cut shows the degrees of compression to which the pith may be subjected. Figure 1 shows a piece of pith (with a string passed through it by means of a needle) cut to the size, to which the cervical canal in a given case may be desired to be dilated. Compressed by the thumbs and fingers, it is reduced to the size of figure 2. The cut represents the actual size and preparation of a tent. To reduce figure 1 to figure 2 required *one minute and a half* by the watch. After introduction into the cervical canal, figure 2 will expand to the size of figure 1. More rapid expansion may be had by pricking the surface of the compressed tent, or by forming a canal through the tent by first inserting a wire through the length of the pith before compressing it.

Fig. 1 Fig. 2

In speaking of sponge tents, Dr. Sims declares he never uses them if he can possibly avoid doing so.

Indeed, so dangerous does he regard them, and so necessary, if possible, is it to find a substitute for them, that he asserts that "he who will give us an efficient, safe, and cheap substitute for sponge tents will confer a great boon upon surgery."

I offer the cornstalk tent as a "safe and cheap substitute." It remains to be seen if it shall, in the hands of the profession, prove "efficient." As above stated, I have used this tent for the last seven years, testing it before giving it to the profession. During this time, I have not had a single accident from its use, and have introduced it many hundreds of times.

Its advantages I will enumerate as follows:

It dilates effectually, but not too rapidly.

It is smooth, soft, and can be removed without force.

It produces no lacerations, abrasions, or irritation of the mucous membrane.

It can be medicated with any substance as easily as the sponge or cloth tent.

It is of vegetable origin, and, hence, does not become putrid and poisonous to the patient.

It may be retained, non-compressed, for days, without injurious results, if no pain occurs.

A number of small tents, filling up the cervical canal, may be used for more rapid expansion.

It can be prepared, in a few minutes, of any desired curve, size, and length.

Any degree of compression may be given it, or it may be used without compression.

It may be perforated, like the sea-tangle, and its power of absorption increased, by pricking its surface.

It will not break upon introduction in the cervical canal; after introduction, it absorbs the secretions, and can be bent without breaking upon removal.

The introduction of the cornstalk tent is usually no difficult matter. I introduce it before or after inserting the speculum, but prefer the latter method as a rule. After bringing the os into view, by means of the speculum (which can easily be done, except in cases of anteversion, when the tenaculum will aid in bringing it into view). the tent is carried to the os uteri, affixed to a stick eight or ten inches in length, by means of a needle fixed in the end of the stick. Holding the small end of the tent in the os, the uterine probe, or a small rod, is placed firmly upon the tent, near the point of insertion of the needle. The stick, in which the needle is fixed, is withdrawn, and the tent pushed gently, but firmly, up the cervical canal by the probe. Between the end of the tent and the probe, the latter being held firmly against the tent, a kind of universal joint is formed, permitting the tent to take the surest and easiest direction into the uterus. Frequently, the uterus ascends before the tent (especially if a little too large), as it is being pressed into the cervical canal, and, in straightening the canal, where there exist curvatures from flexions or versions, the probe end of the tent falls back upon the posterior wall of the vagina. The speculum is withdrawn an inch or so, while the probe, with the tent almost at a right angle with it, lifts the tent into the cervical canal, out of sight, where it is left, a string having been attached to it, by which it may be withdrawn. I endeavor always to carry the large end (probe end) of the tent a short distance within the os uteri. When this is done, it is less liable to escape from the canal into the vagina. The size of the tent should always admit of easy introduction. Slight force will, however, do no harm. It is well to place a packing of cotton, with glycerine, around and upon the os uteri before removing the speculum.

As stated, I allow the patient to withdraw the tent when not used as a dilator. The physician, in removing the tent, should do so with the fingers, and never through the speculum, as air may be admitted to the uterine cavity, and bad results follow.

Typical cases are given in the paper submitted to the association. A pamphlet, containing twenty-five pages, upon uterine tents would doubtless present other points of interest to the readers of the RECORD, but its motto being "*Quicquid Præcipies Esto Brevis,*" I forbear to occupy more space.

## Figures and Tables

**Fig. 1 f1:**
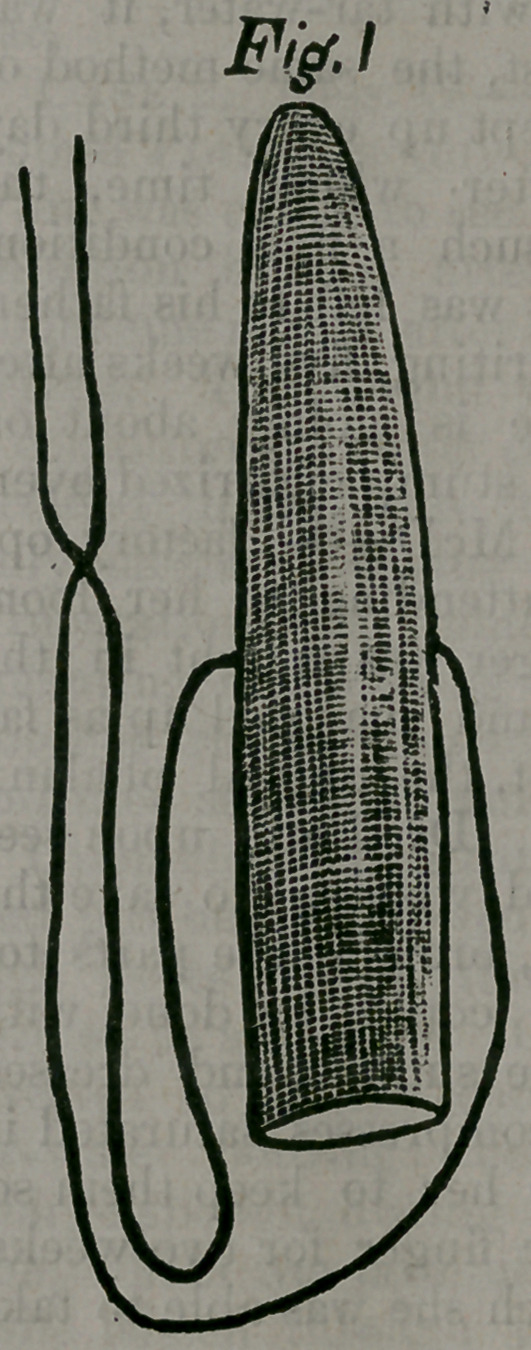


**Fig. 2 f2:**